# *In situ, in vivo*, and *in vitro* approaches for studying AMR plasmid conjugation in the gut microbiome

**DOI:** 10.1093/femsre/fuac044

**Published:** 2022-11-07

**Authors:** Celia Kessler, Jingping Hou, Onalenna Neo, Michelle M C Buckner

**Affiliations:** Institute of Microbiology and Infection College of Medical and Dental Sciences Biosciences Building University Road West University of Birmingham, B15 2TT, United Kingdom; Institute of Microbiology and Infection College of Medical and Dental Sciences Biosciences Building University Road West University of Birmingham, B15 2TT, United Kingdom; Institute of Microbiology and Infection College of Medical and Dental Sciences Biosciences Building University Road West University of Birmingham, B15 2TT, United Kingdom; Institute of Microbiology and Infection College of Medical and Dental Sciences Biosciences Building University Road West University of Birmingham, B15 2TT, United Kingdom

**Keywords:** antimicrobial resistance (AMR), bacteria, plasmid, conjugation, gut microbiome, microbiota

## Abstract

Antimicrobial resistance (AMR) is a global threat, with evolution and spread of resistance to frontline antibiotics outpacing the development of novel treatments. The spread of AMR is perpetuated by transfer of antimicrobial resistance genes (ARGs) between bacteria, notably those encoded by conjugative plasmids. The human gut microbiome is a known ‘melting pot’ for plasmid conjugation, with ARG transfer in this environment widely documented. There is a need to better understand the factors affecting the incidence of these transfer events, and to investigate methods of potentially counteracting the spread of ARGs. This review describes the use and potential of three approaches to studying conjugation in the human gut: observation of *in situ* events in hospitalized patients, modelling of the microbiome *in vivo* predominantly in rodent models, and the use of *in vitro* models of various complexities. Each has brought unique insights to our understanding of conjugation in the gut. The use and development of these systems, and combinations thereof, will be pivotal in better understanding the significance, prevalence, and manipulability of horizontal gene transfer in the gut microbiome.

## Teaser

A discussion of the *in situ, in vivo* and *in vitro* approaches used to understand how antimicrobial resistance spreads by conjugation between bacteria in the human gut microbiome.

## Introduction

The human gastrointestinal (GI) tract contains the majority of the bacterial symbionts that inhabit the human body, with numbers ranging from less than 10^3^ ml^−1^ in the stomach and duodenum, to 10^11^–10^12^ ml^−1^ in the colon (Sekirov et al. 2010). The gut microbiota refers to the sum of the microorganisms in this environment, consisting of a complex and dynamic community structure of mutualistic, commensal, and parasitic symbionts. These, along with their genomes and the surrounding environment, are collectively known as the gut microbiome (Marchesi and Ravel 2015). Generally, the microbiota benefits the host by assisting with intestinal maturation, immunomodulation, and restricting growth and colonization of pathogens by competitive exclusion (Sekirov et al. 2010). However, in addition to traditional ‘pathogens’, opportunistic pathogens, such as *Klebsiella pneumoniae* and extraintestinal pathogenic *Escherichia coli*, can also cause serious infections (Price et al. 2017), and are becoming increasingly difficult to treat owing to the rise of antimicrobial resistance (AMR). Of particular concern are strains such as carbapenem-resistant Enterobacteriaceae (CRE) and extended-spectrum beta-lactamase (ESBL)-producing strains, which are classified as high or critical priority for new drug development by the World Health Organization (Logan and Weinstein 2017, Tacconelli et al. 2018, Cassini et al. 2019).

Bacteria can become resistant to antibiotic therapy by spontaneous mutation or acquisition of antimicrobial resistance genes (ARGs) by horizontal gene transfer (HGT). Conjugation, the transfer of plasmids, provides the bulk of ARG transfer, with clinically relevant ARGs frequently associated with conjugative or mobilizable plasmids (Carattoli 2009, San Millan 2018). The gut microbiome has been identified as a ‘melting pot’ for gene exchange, generally understood to be because of its dense biofilm environment and relatively frequent exposure to antibiotics (Huddleston 2014, Zeng and Lin 2017, Neil et al. 2021a). Strains encoding ARGs in the microbiome are clinically significant, as they have been shown to act as ARG reservoirs (Howden et al. 2013, Husain et al. 2014, van Schaik 2015). Despite ARG transfer to opportunistic pathogens being considered relatively rare events, it is considered a significant factor in the emergence of multidrug resistant strains (van Schaik 2015). Furthermore, successful plasmid transfer events can result in: plasmid rearrangements and evolution leading to multidrug resistance (MDR), prevalence of ARG in multiple species, interactions with small plasmids present in recipient strains, and the potential for convergence of ARGs and virulence plasmids (Barry et al. 2019, Lam et al. 2019, Mathers et al. 2019, Jordt et al. 2020, Stoesser et al. 2020). Plasmid transmission events are of great clinical importance; Marimuthu et al. (2022) found that nearly half (44.8%) of 779 patients who acquired a carbapenem-resistant infection while in hospital were due to plasmid transmission events. Particular emphasis is drawn to bacterial donor strains of foodborne origin, as animal husbandry is a common entry point for resistant strains into the human gut (Bourgeois-Nicolaos et al. 2006, Sparo et al. 2012, Lambrecht et al. 2019).

We are only beginning to understand the effects of the microbiome on bacterial conjugation. Research into this particular area is challenging due to the complex, dynamic, and spatially diverse nature of the gut microbiome, as well as the diversity and plasticity of plasmids. Understanding how conjugative plasmids and their host strains behave in the human gut is paramount to efforts for reducing ARG prevalence and the emergence of new AMR strains in these environments. In this review, we focus specifically on three key approaches (Table 1): first, on direct observation or inference of conjugation *in situ* within the human gut, using case studies from clinical settings. Next, the use of *in vivo* model hosts to investigate conjugation is explored, and finally the potential of *in vitro* models of the gut, which, though the most reductionist and least explored out of the models discussed, show much promise for novel research going forward. Investigating and understanding the factors by which plasmid-mediated transfer of AMR is affected by the gut microbiome and its components is key to designing interventional strategies to tackle this phenomenon.

**Table 1.** List of the approaches discussed and examples of their applications in investigating plasmid conjugation.

Approach	Model type	Donor strain(s)	Recipient strain(s)	Plasmid(s)	Reference
*In situ*	Human experimental	*E. coli*	*E. coli*	Unspecified	Smith (1969)
		*E. coli* K12	Resident *E. coli*	Unspecified	Anderson (1975)
		*E. coli*	*E. coli* K12	Unspecified *sul2*- and *aac(3)-IV*- encoding	Trobos et al. (2008)
		*E. faecium* strain 9730129	*E. faecium* BM4105-RF	Unspecified *vanA-, erm*(B)-, and *vat*(E)- encoding	Lester et al. (2006)
	Human case studies	Resident *Salmonella enterica* serovar Enteritidis or *E. coli*	Resident *S. enteritidis* or *E. coli*	Unspecified	Balis et al. (1996)
		Resident *E. coli*	Resident *E. coli, E. coli* DH5α (*in vitro)*, isolated *E. coli* serotype O15:K52:H1 (*in vitro*)	pNK29	Karami et al. (2007)
		Resident *K. pneumoniae* ST258	Resident *E. coli*, isolated *E. coli* 7364 (*in vitro)*	pKpQIL	Goren et al. (2010)
		Resident *K. pneumoniae*	Resident *Enterobacter aerogenes*	Unspecified *bla*_KPC-2_- encoding	Galani et al. (2013)
		Resident *K. pneumoniae* ST14	Resident *E. coli* ST666, *E. coli* J53 and RuSR (*in vitro*)	Unspecified IncL/M-type *bla*_OXA-48_-encoding	Göttig et al. (2015)
		Resident *E. coli*	Resident *Shigella sonnei*, isolated *S. sonnei* (*in vitro), E. coli* K12 (*in vitro)*	Unspecified ESBL-encoding	Rashid and Rahman (2015)
		Resident *Achromobacter xylosoxidans*	Resident *K. pneumoniae, E. coli* ME8067 and ME8568 (*in vitro*), isolated *K. pneumoniae* ST29 (*in vitro*)	pKUN4507_1	Yamamoto et al. (2016)
		Resident *K. pneumoniae*	Resident *Enterobacteriaceae*	pKPC-e4b7, pKPC-610e/pKPC-5fbf	Conlan et al. (2019)
		Resident *Enterobacteriaceae*	Resident *Enterobacteriaceae, E. coli* J53 (*in vitro*)	Unspecified IncL/M-type *bla*_OXA-48_-encoding	León-Sampedro et al. (2021)
*In vivo*	Mice with unmodified microbiota	*E. coli* MDS42	*E. coli* MDS42	IncF-type pOX38 and IncW-type R388	Palencia-Gándara et al. (2021)
		*E. coli* Nissle1917	*E. coli*	pCURE-K-307	Lazdins et al. (2020)
	Diassociated mice	*E. faecalis*	*E. coli*	pAT191	Doucet-Populaire et al. (1992)
		*E. faecalis* OG1S	*E. faecalis* OG1RF or OG1ES	pCF10	Hirt et al. (2018)
		*E. faecium* E462 and UW7	*E. faecalis* JH2-2	Unspecified *vanA* and *tet*(L)-encoding and unspecified *vanA, tet*(L), *erm*(B), and *tet*(M)- encoding	Bourgeois-Nicolaos et al. (2006)
		*E. coli* UB1832	*E. coli* PG1	R388	Maisonneuve et al. (2002)
	Diassociated rats	*L. plantarum* DG 522 or DG 507	*E. faecalis* JH2-2	Unspecified *tet*(M)-encoding or unspecified *tet*(M) and *erm*(B)- encoding	Jacobsen et al. (2007)
	ASF-associated mice	*K. pneumoniae* ST14	*E. coli* J53 and RuSR	Unspecified IncL/M-type *bla*_OXA-48_-encoding	Göttig et al. (2015)
		*S*. Kentucky CVM29188	*E. coli* HS-4 and MGN026	pCVM29188_146	Ott et al. (2020)
	HFA mice	*E. faecalis* OG1S	*E. faecalis* OG1RF or OG1ES	pCF10	Hirt et al. (2018)
		*E. faecium* E462 and UW7	*E. faecium* 64/3 or *E. faecalis* JH2-2	Unspecified *vanA* and *tet*(L)-encoding and unspecified *vanA, tet*(L), *erm*(B) and *tet*(M)- encoding	Bourgeois-Nicolaos et al. (2006)
		*E. coli* UB1832	*E. coli* PG1	R388	Maisonneuve et al. (2002)
		*E. faecium* HC-VI2	*E. faecium* 64/3 or *E. faecalis* JH2-2	Unspecified *vanA*-encoding	Mater et al. (2005)
	HFA rats	*S*. Virchow 3464b	*E. coli* J5	Unspecified *bla*_CTX-M-9_-encoding	Faure et al. (2010)
	Antibiotic-treated mice	*S*. Typhimurium SL1344	*E. coli* 8178	Unspecified IncI1-type *cib*-encoding	Stecher et al. (2012)
		*E. coli* ST117, ST648, ST40, ST69, ST80, ST95, ST6697, and ST131	*E. coli* 8178, unspecified *E. coli* isolates and *S*. Typhimurium 14028	Various ESBL-encoding	Benz et al. (2021)
		*E. coli* SF-166	*S*. Typhimurium SL1344	pESI	Aviv et al. (2016)
		*S*. Heidelberg SL-312, *S*. Bredeney, and *E. coli* O80:H26	*E. coli* CV601gfp and resident *E. coli* O2:H6	Various *bla*-encoding	Laskey et al. (2020)
		*E. faecalis* OG1SS	*E. faecalis* T11RF	pAM714 and pAM771	Price et al. (2019)
		*E. coli* Nissle 1917	*E. coli* Nissle 1917 and *C. rodentium* DBS100	TP114	Neil et al. (2021b)
		*E. faecalis* CS19	*E. faecalis* JH2-SS	Unspecified	Sparo et al. (2012)
		*S*. Typhimurium SL1344	*S*. Typhimurium ATCC 14028s, *E. coli* 8178	P2	Bakkeren et al. (2019)
		*S*. Typhimurium SL1344	*S*. Typhimurium ATCC 14028s	P2	Bakkeren et al. (2021)
		*S*. Typhimurium ATCC 14028s	*S*. Typhimurium ATCC 14028s	P2	Bakkeren et al. (2022)
	Antibiotic-treated Syrian hamster	*E. faecalis* FA2-2	*E. faecalis* JH2-SS	pADl	Huycke et al. (1992)
	*D. melanogaster*	*E. coli* MG1655	*E. coli* HS-4	pKJK5-GM, pCVM29188_146, pC20-GM	Ott et al. (2021)
	*G. mellonella* larvae	*K. pneumoniae* ST14	*E. coli* J53 and RuSR	Unspecified IncL/M-type *bla*_OXA-48_-encoding	Göttig et al. (2015)
*In vitro*	Mating experiments	*E. coli* MG1655	*E. coli* MG1655	Unspecified IncI1 *bla*_CTX-M-1_-encoding	Duxbury et al. (2021)
		*S*. Infantis 119 944	*E. coli* ORN172	pESI	Aviv et al. (2016)
	Single-vessel chemostat	*E. coli* B1-54	*E. coli*	Unspecified IncI1-type *bla*_TEM-52_-encoding	Smet et al. (2011)
	Multivessel chemostat	*K. pneumoniae* ST147 and ST661	Resident *E. coli*	Unspecified IncFIB/IncFII *bla*_NDM-1_-encoding, pKpQIL-D2	Rooney et al. (2019)
	M-SHIME	*E. coli* MB6212	Resident coliforms	p5876	Lambrecht et al. (2019)
		*E. coli* MB6212	Resident coliforms	p5876	Lambrecht et al. (2021)
	PolyFermS	*E. faecalis* CG110	Resident *E. avium*	pRE25*	Haug et al. (2011)

## In situ

### Observational and follow-up studies

Due to its spatially diverse microbiological niches, complex array of symbionts and the impact of host factors such as the immune system, the human GI tract is notoriously difficult to reproduce experimentally. As a result, the value of *in situ* experiments and observations are difficult to rival with models. Early experiments involving oral administration of donor and recipient *E. coli* to human volunteers demonstrated detectable levels of transmission of AMR phenotypes, though accompanied by very little colonization (Smith 1969, Anderson 1975). More recent studies have also demonstrated transfer of AMR genes in the intestines of human volunteers, including sulphonamide resistance between human-derived *E. coli* strains (Trobos et al. 2008), and vancomycin resistance between animal- and human-derived *Enterococcus faecium* (Lester et al. 2006). These have since generally given way to animal and laboratory models due to the ethical risks of colonizing subjects with AMR strains.

Observational studies can utilize faecal samples or rectal swabs, most frequently from hospitalized patients where faecal screening is often routine (Goren et al. 2010, Galani et al. 2013, Conlan et al. 2019, Prevel et al. 2019, León-Sampedro et al. 2021), to investigate the abundance and epidemiology of AMR bacteria. Accounts of nosocomial outbreaks of AMR pathogens are abundant in the literature, with identified plasmid-borne ARGs often characterized *in silico* and *in vitro*. In many cases, the potential for plasmid dissemination is assumed, given the ability of the plasmid to conjugate in subsequent *in vitro* experiments using isolated strains (Leavitt et al. 2010, Aghamohammad et al. 2019, Bocanegra-Ibarias et al. 2019). More direct evidence of conjugation events can come from cases where multiple samples are taken from the same patient or hospital, either within nosocomial outbreaks (Galani et al. 2013, Göttig et al. 2015) or in nonoutbreak contexts (Balis et al. 1996, Karami et al. 2007, Goren et al. 2010, Rashid and Rahman 2015, Yamamoto et al. 2016, León-Sampedro et al. 2021). These conclusions can be drawn from the isolation of separate strains carrying the same plasmid either simultaneously (Balis et al. 1996, Rashid and Rahman 2015, Conlan et al. 2019), or sequentially, where a plasmid can be hypothesized to have transferred between two sampling dates (Karami et al. 2007, Goren et al. 2010, Galani et al. 2013, Göttig et al. 2015, Yamamoto et al. 2016, León-Sampedro et al. 2021).

*In vitro* conjugation experiments using *E. coli* recipients (usually laboratory strains such as K12, J53, or DH5α) were used in many of the cited studies to test the potential of these plasmids to conjugate (Karami et al. 2007, Göttig et al. 2015, Rashid and Rahman 2015, Yamamoto et al. 2016, León-Sampedro et al. 2021). Alternatively, or in addition to laboratory strains, some studies use susceptible isolated strains as recipients (Karami et al. 2007, Goren et al. 2010, Yamamoto et al. 2016). While use of such ‘real-world’ recipient strains can be challenging, it more closely models the recipients encountered in the gut. In these studies, successful conjugation was taken as supporting evidence for the occurrence of conjugation in the gut. In some cases, however, conjugation was unsuccessful (Goren et al. 2010, Galani et al. 2013). A suggested explanation was the possibility of indirect conjugation through an intermediate strain (Galani et al. 2013), though natural transformation of plasmid DNA in the gut microbiome is theoretically also possible but has not yet been characterized (McInnes et al. 2020). It is also evident that using different strains and *in vitro* conditions does not exactly replicate the putative *in situ* event (Hardiman et al. 2016) as Goren et al.(2010), using their isolate as a recipient, were unable to observe any conjugation *in vitro*. It is also plausible that these conjugative events in the gut are rare and, therefore, below the limit of detection of an *in vitro* assay. But these events may provide a fitness advantage, especially in a hospital setting where selective pressure from antibiotic use is high. This is one of the key advantages of *in situ* studies; they find even extremely rare events, which for a variety of reasons such as AMR or virulence, are selected for in the patient. Such rare but ‘beneficial’ events can then contribute to the rise of globally successful AMR strains. Indeed, *in situ* studies are key for monitoring the evolution of and understanding the epidemiology of emergent bacteria of clinical concern, but do not permit the same level of experimentation that the other models discussed below allow.

The apparent sampling bias in the cited case studies favouring Enterobacteriaceae, especially CRE, is likely a tribute to a combination of factors: hospitals routinely screening for carbapenem-resistant organisms (Goren et al. 2010, Galani et al. 2013, Logan and Weinstein 2017, Tacconelli et al. 2018, León-Sampedro et al. 2021), and the propensity of Enterobacteriaceae to carry clinically relevant AMR plasmids (Carattoli 2009), reside as opportunistic pathogens in the gut (van Schaik 2015, Price et al. 2017) and cause nosocomial infections (Vincent 2003). More research is needed to understand *in situ* transmission in other bacteria.

### Methods used in *in situ* studies

Faeces or rectal swabs are the most common and pragmatic samples taken as a proxy for the microbiota in *in situ* studies (Smith 1969, Anderson 1975, Lester et al. 2006, Trobos et al. 2008, Göttig et al. 2015, McInnes et al. 2020). However, these subject samples to a certain bias whereby the composition is representative of only the endpoint microbiota. Microbiota associated with different niches are phylogenetically distinct (Rangel et al. 2015, Ringel et al. 2015), suggesting that faeces may be a poor proxy for microbiome representation higher along the GI tract. Additionally, there is no standard protocol for the handling of faecal samples, with a trade-off between homogenizing to reduce variation and preserving sample community structure (Tang et al. 2020). Most alternative methods, e.g. biopsies and other surgical approaches, are invasive and their suitability for gut microbiota analysis of healthy participants disputed (Tang et al. 2020), though are used in some studies relating to the microbiome (Ringel et al. 2015, Tap et al. 2017).

The rapid development of novel bioinformatic approaches in recent years has improved the quality of culture-independent data available from microbiome samples, such as abundance of ARGs and genomic signatures of HGT. Methods for detecting HGT events in metagenomes are reviewed by Douglas and Langille (2019), with their specific applications in the gut microbiome reviewed by McInnes et al. (2020). Of particular interest are methods, which link ARGs to their plasmid and microbial hosts such as epicPCR or 3C, which vastly improve insight into the genomic contexts of individual ARGs, and have the potential for assessing transmissibility and monitoring transmission events (McInnes et al. 2020, Yaffe and Relman 2020). Overall, the importance of genomics for detecting and characterizing HGT events *in situ* cannot be overstated. For example, analysis of sequencing data from carbapenem-resistant *K. pneumoniae* across Europe revealed three unique and successful approaches to dissemination: success of a single plasmid encoding ARGs, success of a specific clone, or transient association with a range of plasmids (David et al. 2020). However, surveillance alone is not sufficient to address the many fundamental biological questions, which still surround plasmid transmission and dynamics within the microbiome. This is where experimental models, which allow the testing of hypotheses and the introduction of controls, is essential.

## *In vivo* models

Experiments using *in vivo* models allow for informed extrapolations on how plasmids might behave in the human gut microbiome. Rodents, specifically mice, are by far the most used models due to vast amounts of historical research and their firm establishment as models for human health and disease physiology. The benefits and drawbacks to their use in modelling the human gut has been extensively reviewed elsewhere (Nguyen et al. 2015, Hugenholtz and de Vos 2018, Kennedy et al. 2018). Notably, comparative meta-analyses of the murine and human gut microbiota highlight that, while similar at the phylum level, the microbiota differ in the relative abundance of different genera (Krych et al. 2013, Hugenholtz and de Vos 2018), and can be influenced by host factors such as diet and genotype (Elzinga et al. 2019). Despite these differences and subsequent limitations in translatability, mice with naturally occurring microbiota are insightful models for studying plasmid transmission. As an example, Palencia-Gándara et al.(2021) demonstrated that synthetic fatty acid 2-hexadecynoic acid reduced conjugation of an IncF and IncW plasmid between laboratory *E. coli* strains 50-fold in the intestines of C57BL/6 mice. Other examples include elucidating the role of conjugation in bacteriocin-producing Enterococci (Kommineni et al. 2016), and determining the *in vivo* antibiotic selection dependence of a plasmid displacement vector for reducing the prevalence of an IncK plasmid in *E. coli* within BALB/c mice (Lazdins et al. 2020).

However, due to the fundamental differences between the murine and human microbiota, conventional mice may not always be the most appropriate model for simulating the human gut. The following sections review applications of studying plasmid conjugation using diassociated mice, mice inoculated with defined microbiota, and mice with microbiota manipulated by antibiotics. Non-murine models have occasionally been used to model plasmid conjugation in the human gut microbiome, and are briefly described.

### Diassociated mice

Inoculation of germ-free mice with known microbes (gnotobiotic mice) results in high levels of colonization by the inoculated organisms due to the elimination of competition usually imposed by endogenously colonizing bacteria, with inoculants detected in faeces reaching 10^9^–10^10^ CFU g^−1^ (Schjørring and Krogfelt 2011). This also guarantees colonization by the inoculated strains, whereas mice with resident microbiota tend to display colonization resistance (Mater et al. 2005, Bourgeois-Nicolaos et al. 2006, Ott et al. 2020). A donor and recipient strain can be used to inoculate germ-free mice, creating the diassociated mouse model, where the resulting high concentrations of donor and recipient bacteria improves the chance of contact between strains, thus improving chances of conjugation (Bourgeois-Nicolaos et al. 2006, Schjørring and Krogfelt 2011). This makes this model useful for proof-of-concept studies for conjugation in the gut, however, has otherwise limited translatability due to its reductionist nature, as well as observations of compromised animal health such as lowered immune function (Round and Mazmanian 2009).

Examples of applications of the diassociated model include demonstrating conjugation between Gram-positive and -negative species in the gut in the absence of antibiotic selection (Doucet-Populaire et al. 1992), where plasmid pAT191 from *Enterococcus faecalis* was observed to transfer to *E. coli* by the detection of low amounts of transconjugants in C3H mouse faeces. However, absence of transconjugants isolated from the intestines at autopsy suggested that the *E. coli* transconjugants did not colonize the murine intestine well. Hirt et al. (2018) compared *in vivo* transmission of the pheromone-inducible plasmid pCF10 between *E. faecalis* strains in Swiss Webster mice, where inoculation of donors with pheromone-producing recipients resulted in significantly higher transmission rates than in pheromone-inactivated recipients. However, coculture with both recipients did not rescue pCF10 conjugation into pheromone-inactivated recipients as it did *in vitro*, implicating a role of the intestinal microenvironment, such as spatial distribution, in this discrepancy (Hirt et al. 2018). Use of the diassociated model has also been compared with *in vitro* studies and other murine models later discussed, to compare the dynamics of plasmids in different models (Maisonneuve et al. 2002, Bourgeois-Nicolaos et al. 2006, Hirt et al. 2018).

### Mice with a defined microbiota

An increasingly popular approach is to inoculate germ-free mice with a defined microbiota of multiple strains, resulting in a model closer to physiological relevance but maintaining a low enough complexity to investigate elements such as host factors, with limited confounding effects from the resident microbiota. Established defined microbiota exist for these purposes, which have been each tailored to address unique questions. The most studied defined microbiota, including the altered Schaedler flora (ASF), Oligo-MM^12^ and SIHUMIx have been reviewed elsewhere, in addition to other communities and their applications (Elzinga et al. 2019).

The ASF is made up of eight microorganisms that were initially isolated from the murine microbiota, as defined by Dewhirst et al. (1999). Free of Enterobacteriaceae, ASF mice can be easily colonized by strains of Enterobacteriaceae of interest, making this a highly useful model for studying this clinically important family. For example, C57BL/6 ASF-colonized mice were simultaneously inoculated with *bla*_OXA-48_-carrying *K. pneumoniae* of clinical origin as a donor, and *E. coli* J53 or the slow growing, motile-deficient *E. coli* RuSR as recipients, to support investigations into the clinical plasmid transmitting *in situ* (Göttig et al. 2015). Conjugation frequency as determined by J53 transconjugants isolated from caecal contents was nine times that of *in vitro* liquid mating. However, though RuSR transconjugants were detected *in vitro*, none were isolated *in vivo*, indicating a role for recipient fitness and motility in conjugation *in vivo* (Göttig et al. 2015). Ott et al. (2020) compared the proinflammatory environment of interleukin-10-deficient against wild-type 129S6/SvEv ASF-colonized mice, to study the effect of host factors on the transfer of plasmid pCVM29188_146 from *Salmonella enterica* serovar Kentucky into *E. coli*, observing no difference in transconjugant yields. When investigating different mouse genetic backgrounds, conjugation frequency was lower with C3H/HeN ASF-colonized compared to 129S6/SvEv ASF-colonized mice, correlating with a depletion in some of the microbiota members such as Clostridia in the C3H/HeN mice over the course of the 28-day experiment (Ott et al. 2020), providing evidence that mouse genetic background can affect microbiota composition, which in turn impacts conjugation.

Alternatively to the ASF, the Oligo-MM^12^ microbiota consists of 12 strains and was designed to provide colonization resistance against *S. enterica* serovar Typhimurium (Brugiroux et al. 2016). Benz et al. (2021) used Oligo-MM^12^-inoculated C57BL/6 mice to demonstrate that trends in conjugation efficiency relating to plasmid, donor and recipient combinations, translated well between *in vitro* liquid mating conditions and *in vivo*, when studying transfer of IncI and IncF ESBL-encoding plasmids from clinical *E. coli* strains to *E. coli* and *S*. Typhimurium recipients.

The human flora-associated (HFA) model refers to germ-free mice that have been inoculated with human faecal bacteria. Although the closest model to human application due to the humanized element of the microbiota, genetic, and feeding discrepancies result in modulation of the microbiota in mice (Schjørring and Krogfelt 2011). Despite being a popular model in GI research, it has only a handful of applications in studying conjugation in the human gut. Studies comparing conjugation in HFA mice with diassociated mice have reported different outcomes, with outcome of the diassociated model tending to be closer to that of *in vitro* mating experiments (Maisonneuve et al. 2002, Bourgeois-Nicolaos et al. 2006, Hirt et al. 2018). HFA C3H/He mice fed different diets demonstrated that dairy products such as yoghurt and milk reduced the conjugation frequency of the *E. coli* plasmid R388 to an inoculated *E. coli* recipient (Maisonneuve et al. 2002). Lactose prevented detection of any transconjugants in HFA mice, but did not have an effect on conjugation in diassociated C3H/He mice (Maisonneuve et al. 2002). Other studies investigating transfer of *vanA*-carrying plasmids between different species of Enterococci have reported successful intraspecies conjugation between *E. faecium* strains, but no conjugation to *E. faecalis* recipients when tested in HFA C3H (Bourgeois-Nicolaos et al. 2006) and C3H/He (Mater et al. 2005) mice. Bourgeois-Nicolaos et al. (2006) observed a discrepancy between the HFA and diassociated C3H models, where transfer to *E. faecalis* was observed in diassociated mice as well as in *in vitro* filter mating experiments, but not in HFA mice. Discrepancies such as this likely arise from the artificially high densities of donor/recipient bacteria in the diassociated model, which relates more closely to *in vitro* mating assays, but reduces the extrapolations that can be made from this model. In support of this, establishment of just a four-strain defined microbiota in Swiss Webster reduced conjugation of a pheromone-inducible plasmid between *E. faecalis* strains in the colonized lower intestinal tract, with no effect on *E. faecalis* populations, in comparison with diassociated Swiss Webster mice (Hirt et al. 2018).

### Antibiotic-treated mice

The antibiotic-treated mouse model is characterized by the depletion of select bacterial species (Hentges et al. 1984, Schjørring and Krogfelt 2011, Kennedy et al. 2018). Antibiotic treatment and subsequent bacterial depletion reduce the colonization resistance to inoculated strains (Hentges et al. 1984, Stecher et al. 2006, Gumpert et al. 2017, Laskey et al. 2020). It can provide useful disease models, e.g. *S*. Typhimurium infection in untreated mice results in a systemic typhoid-like infection, but in streptomycin-pretreated mice results in colitis as it would in humans (Barthel et al. 2003). Using this colitis model, Stecher et al. (2012) coinfected C57BL/6 mice with *E. coli* and *S*. Typhimurium resulting in a rapid ‘bloom’ of both strains and high plasmid transfer rates compared to controls infected with avirulent *S*. Typhimurium. Further investigations using the avirulent donor and ASF mice, to aid colonization of Enterobacteriaceae, determined that it was the densities of donor and recipient, rather than the inflammation directly, that promoted conjugation (Stecher et al. 2012).

In a series of work exploring HGT in *S*. Typhimurium, Bakkeren et al. (2019) used streptomycin-pretreated *Nramp1^+/+^* mice (Stecher et al. 2006) to enable robust colonization of strains of interest and explore the role of *Salmonella* persisters’ contribution to HGT (Bakkeren et al. 2019). They showed that persister populations from systemic organs reseeded the gut lumen, where they transferred a native *Salmonella* plasmid, P2, to inoculated recipients (*S*. Typhimurium and *E. coli*) at very high frequencies. Importantly, they found that reseeding of the lumen was the rate-limiting step and evolutionary bottleneck, rather than plasmid conjugation. This demonstrated the importance of using *in vivo* models to study conjugation in the gut, as this work showed the contribution of persisters from nongut tissues to act as plasmid donors in the gut (Bakkeren et al. 2019). Later, Bakkeren et al. (2019) showed that during persistent *Salmonella* infection, tissue reservoirs of *Salmonella* (not persister cells), are also able to reseed the gut, receive a conjugative plasmid, then invade back into the mouse tissues. As previously, reseeding rather than conjugation was the rate limiting step. Remarkably, this work also demonstrated that donor bacteria carrying ESBL-encoding plasmids were able to reduce the concentration of a β-lactam antibiotic in the gut lumen enough that β-lactam sensitive *S*. Typhimurium recipients were able to reseed the gut and acquire the plasmid, demonstrating cooperation between the donor and recipient bacteria. Further exploring cooperation and HGT, Bakkeren et al. (2022) used the same native *Salmonella* plasmid, this time carrying a virulence regulator, *hilD*, and demonstrated that conjugation into a *hilD* mutant restored virulence, but at a fitness cost. Indeed, mutant ‘cheaters’ with inactivated *hilD* rapidly arose in the gut lumen. Together these eloquent studies demonstrate the importance of the antibiotic-treated mouse model in expanding our understanding of not only HGT, but also infection, virulence, bacterial evolution, and bacterial cooperation.

Aviv et al. (2016), also used a streptomycin-pretreated model with C57/BL6 mice and observed conjugation into the inoculated *S*. Typhimurium strain. This was a secondary conjugation event, the donor strain being a mouse commensal *E. coli* transconjugant isolated from an earlier mouse conjugation experiment, demonstrating the transfer of an AMR plasmid between commensal and pathogenic strains in the context of infection, and significance of the commensal microbiota as a reservoir of AMR plasmids that can transmit into pathogenic bacteria.

Inflammation induced by a combination of ampicillin treatment and coinfection with multiple β-lactam-resistant Enterobacteriaceae was studied in C57BL/6 mice, with *S*. Heidelberg, *S*. Bredeney, and *E. coli* O80:H26 inoculated as donors and an *E. coli* recipient. Despite ampicillin treatment, when inoculated alone the *E. coli* donor strain did not appear to colonize nor conjugate its β-lactam resistance-encoding plasmid (Laskey et al. 2020). Colonization of *E. coli* was successful in coinfection with both *Salmonella* strains, however, and the IncI2 plasmid was further detected in both the inoculated recipient *E. coli* and an endogenous *E. coli* strain. Induced inflammation, as well as observed dysbiosis seemed to promote conjugation in this case, similarly to other accounts. Price et al. (2019) treated C57BL/6 mice with streptomycin, gentamicin, and erythromycin to simulate antibiotic-induced dysbiosis, and investigated conjugation of pheromone-responsive plasmids between strains of *E. faecalis*, where the recipient strain expressed a CRISPR-Cas system targeting the plasmids. CRISPR-Cas expression almost entirely prevented plasmid acquisition *in vivo*, but significantly less so *in vitro* in both liquid and agar biofilm mating. This highlights the role of *in vivo* studies, where the additional pressures on bacteria provided in a host animal significantly alter the outcome, with implications that CRISPR-Cas could be sufficient to prevent HGT in real-world settings, even if insufficient *in vitro*, although more work is needed to confirm this. Conjugative delivery of a CRISPR-Cas-encoding plasmid has also been shown to successfully target drug-resistant *E. coli* and *Citrobacter rodentium* in streptomycin-treated C57BL/6 mice (Neil et al. 2021b).

Another example of antibiotic use in murine models to aid in colonization and detection of strains of interest, used streptomycin treatment to facilitate colonization of streptomycin-resistant donors and transconjugants (Neil et al. 2020). This study investigated transfer rates of 13 plasmids from 10 different Inc types in agar, broth, and murine infections (Neil et al. 2020). They identified an IncI2 plasmid that conjugated at high rates in the gut, and using transposon mutagenesis determined that the Type IV pilus, which mediates mating pair stabilization, was required (Neil et al. 2020). One of the unique aspects of this work is it allows direct comparison of a range of plasmids, as all plasmids were inserted into isogenic donor/recipient pairs, and tested in multiple models of conjugation. In addition, their high-level conjugating plasmid was IncI_2_, and the plasmid P2 (used by Stecher et al. 2012, Bakkeren et al. 2019, 2021, 2022) was IncI_1_, and also conjugated at very high levels in the gut.

The above methods of generating murine models can be combined. For example Sparo et al. (2012) used ceftriaxone to eliminate coliform bacteria in BALB/c mice before adding human microbiota, to study the transfer of gentamicin resistance between *E. faecalis* strains. Additionally, other mouse models exist with the potential for use in this field, e.g. a recently developed model of occult colonization, which uses treatment with antibiotics to stimulate outgrowth of carbapenem-resistant *K. pneumoniae* (Sim et al. 2022) could be a promising model to study the transmission of antibiotic resistance plasmids.

No one mouse model will fit every purpose. The diassociated model is sufficient to demonstrate that conjugation can occur, and could be useful to study direct host involvement (e.g. host metabolites), but the lack of microbiota and poorly developed immune system makes it a poor model for more in depth studies. Defined microbiota models are particularly useful for monitoring impact of specific strains/bacterial interplay on conjugation, and can provided added complexity to study fitness, in comparison to *in vitro* or diassociated models. One benefit of defined microbiota models are their reproducibility, by using the same specific strains to colonize each mouse. Conversely, the HFA model will have more variability in flora composition, but gives a better picture of what may happen in the human microbiome. The predominant model used to date to study plasmid conjugation and dynamics is the antibiotic-treated mouse, as it allows the high density of donors and recipients required to observe conjugation events. In particular, use of *Salmonella-*based mouse models have regularly been used. *Salmonella* infection models are very well-established in the scientific community, and the intricacies of *Salmonella* virulence are relatively well-understood, which makes it an ideal model to study HGT. However, many of these studies have limited applications beyond *Salmonella* infections, and while *Salmonella* is undoubtedly an important pathogen, the global AMR crisis requires we expand our understanding of HGT in other Gram-positive and -negative bacteria, such as *K. pneumoniae, E. coli, E. faecium*, and *Acinetobacter baumannii*.

### Other animal models

As an alternative rodent model to mice, rat models have been applied to the study of plasmids in the gut but are less common, likely owing to their cost of upkeep (Ericsson and Franklin 2015). Using diassociated Sprague–Dawley rats, Jacobsen et al. (2007) observed rare transfer of *tet(M)*- and *erm(B)*-containing plasmids from *Lactobacillus plantarum* to *E. faecalis*. With HFA rats, Faure et al. (2010) studied plasmid-mediated *bla*_CTX-M-9_ transmission from *S. enterica* serovar Virchow to resident Enterobacteriaceae. Conjugation was successful only when the rats were treated with cefixime, though use of an *E. coli* J53 recipient produced transconjugants in both conditions. Transfer of pheromone-inducible plasmids between strains of *E. faecalis* have also been studied in a Syrian hamster model of Enterococcal overgrowth, facilitated by streptomycin and spectinomycin treatment before inoculation (Huycke et al. 1992), though this model is rarely used in this context. The infrequent use of these models makes the studies difficult to compare to other models, though the findings are in line with conjugation studies in mice.

The fruit fly *Drosophila melanogaster* has been proposed as a reduced complexity model for *in vivo* plasmid transfer, owing to its lack of adaptive immunity and limited diversity and transient nature of its gut microbiota (Ott et al. 2021). This transience limits the duration of conjugation experiments, but Ott et al. (2021) isolated *E. coli* transconjugants from fly homogenates made 1 hour after exposure to the inoculated recipient strain.

Larvae of the wax moth *Galleria mellonella* are a common infection model, generally used in killing assays to determine bacterial virulence, though have been used as a model for plasmid transfer. Göttig et al. (2015) characterized a clinical *K. pneumoniae* donor carrying *bla*_OXA-48_, which alongside *E. coli* J53 or slow growing motile-deficient *E. coli* RuSR recipient, was injected into *G. mellonella* larvae and incubated for 24 hours. Conjugation frequency in J53 was over 100 times higher than *in vitro* liquid mating, and no RuSR transconjugants were detected despite successful conjugation *in vitro*. These observations mirrored those made in ASF mice, supporting the indication of a role for fitness and motility in conjugation *in vivo* (Göttig et al. 2015). That the findings from *G. mellonella* and ASF mice were similar, lends credence to this model, though additional studies to support this are needed. Overall, the use of non-murine models to model the human gut, though few, are interesting and may increase in prevalence. They provide a ‘middle-ground’ in terms of complexity, and are useful in cases where, e.g. a mammalian immune system, is not needed.

## *In vitro* models

Replace; reduce; refine: the ‘3Rs’ of a humane approach to using animals in science (Russell and Burch 1959) are still widely referenced in contexts from research to policy (Tannenbaum and Bennett 2015). National and international policy based around the 3Rs heavily restricts the use of animals in research and actively promotes research into alternative methods (Cronin 2017).

*In vitro* models have the inherent advantage of having far more controllable parameters compared with *in vivo*, though the vast reduction in complexity compared with *in situ* environments compromises the translatability of results. Components are much easier to access, monitor, and sample. Relatively, simple broth or agar mating experiments under different conditions can provide useful insights into plasmid dynamics, e.g. demonstrating that microaerobic conditions were more favourable than either aerobic or anaerobic for conjugation of the megaplasmid pESI in *S. enterica* serovar Infantis; 37°C was more favourable than 27°C, and hydrogen peroxide-induced oxidative stress also promoted conjugation, through increases in pilus gene transcription (Aviv et al. 2016). Alternatively, when supernatant from lactic acid bacteria derived from chicken caecal samples were added to *in vitro E. coli* matings, it did not affect conjugation frequency, however, it did affect growth rates (Duxbury et al. 2021). Alternatively, higher complexity models have been developed with the aim of better mimicking the microbiome *in vitro* than liquid or agar mating experiments. Ultimately, the difficulty of developing and using insentient alternatives to animal research depends on the necessary complexity of the model for the purposes of a particular investigation. *In vitro* models can replicate aspects of bacteria–bacteria interactions, and can even include some host factors. However, they cannot replicate the complexities of the immune system or inflammation and the impacts of this on plasmid dynamics.

### Key features of *in vitro* models

In the context of the gut microbiome, simple models using coculture with microbial communities in multivessel culture systems have been used since the 1980s (Veilleux and Rowland 1981). Recreating the much more complex parameters of a real gut such as host cells and their regeneration, tissue structure, mechanotransduction, peristaltic flow, metabolite gradients, and biochemical host–microbe cross-talk is more difficult, and covered in detail in reviews by Costa and Ahluwalia (2019) and Wang et al. (2018). These reviews are primarily focused on recreating the environmental niche for host gut cells to study factors of host health, such as inflammation, probiotics, or nutrition bioavailability. To study the microbiome itself, features such as mechanotransduction, stem cell differentiation control, and the ability to monitor host cells are less central, highlighting how models differ based on research context. Several of these microbiome models are reviewed by Venema and van den Abbeele (2013), and here we discuss their applications for use in conjugation experiments.

Generally, these models are single- or multicompartmental with vessels simulating environments of the GI tract, either using chemostat or batch fermentation methods. Batch fermentation models are the simplest, cheapest, and most frequently used, however, are far from physiological (Venema and van den Abbeele 2013), and only suitable for experiments lasting up to about 24 hours due to accumulation of inhibitory metabolites and depletion of substrates. This can be prevented by using chemostat bioreactor models, some of which have been successfully used to model the human gut and study plasmid transmission therein (Haug et al. 2011, Smet et al. 2011, Lambrecht et al. 2019), or other gut environments such as the chicken caecum (Card et al. 2017). Conjugation has been observed in a single-vessel chemostat system between an ESBL-producing *E. coli* of broiler origin to *E. coli* originating from the donor microbial slurry (Smet et al. 2011). Common controllable variables in both single- and multivessel chemostat models include pH, anaerobic conditions, agitation, and presence of a stabilized microbial slurry from a human donor. Models often incorporate mucosal components, as mucus has been identified as an important microbial mediator of growth and access to host cells (van den Abbeele et al. 2009), with bacterial populations localized to mucin surfaces shown to be phylogenetically and metabolically distinct from those grown planktonically (Macfarlane et al. 2005).

### Established *in vitro* models

Macfarlane et al. (1989) developed one of the first multivessel chemostat models of the human gut to address the different environments across the proximal and distal colon using three vessels. Adaptations of this model have since been used to demonstrate the conjugation of *bla*_NDM_- and *bla*_KPC-2_-encoding plasmids under antibiotic pressure from a *K. pneumoniae* donor strain to *E. coli* resident in the human faecal slurry used in the model (Rooney et al. 2019).

Also, based on the Macfarlane model, the Simulator of the Human Intestinal Microbial Ecosystem (SHIME®; ProDigest, Belgium) incorporates two additional vessels representing the stomach and small intestine environments (van de Wiele et al. 2015). SHIME® has been further developed into the mucosal SHIME (M-SHIME), containing regularly replaced artificial mucosal components to the vessel walls, simulating a naturally renewing mucus layer (van de Wiele et al. 2015). Using the M-SHIME gut to study conjugation of plasmid p5876 from a broiler chicken-derived *E. coli* strain, Lambrecht et al. (2019) isolated approximately 30 times more transconjugants from mucin microcosms compared to the lumen. This demonstrates the importance of mucosal structures for conjugation, and in light of this *in vitro* models should aim to incorporate such structures in their development.

Further investigation of ARG transmission from foodborne *E. coli* to coliform members of the gut microbiota using the M-SHIME model has explored additional factors such as *E. coli* dosage, donor microbiota, and a single dose of cefotaxime (Lambrecht et al. 2021). Broiler chicken-derived *E. coli* conjugated an IncF plasmid encoding cefotaxime resistance to resident coliforms within hours, and none of the above variables significantly impacted the rate of conjugation, nor the composition of the microbiota as determined by Illumina sequencing of 16S rRNA (Lambrecht et al. 2021).

The PolyFermS model (Cinquin et al. 2004, Zihler Berner et al. 2013), in contrast to mucin microcosms, uses polysaccharide gel beads to immobilize part of the microbiota used in its vessels, increasing microbial density to physiological levels. This model has been used to investigate conjugation, determining that an *E. faecium* donor can transfer a multidrug resistant plasmid pRE25 to members of the commensal microbiota, as demonstrated by isolation of an *Enterococcus avium* transconjugant (Haug et al. 2011). Additional scaffolds have been explored for culture of microbiota samples *in vitro*, such as electrospun gelatin, which has been successfully shown to support microbial growth and preserve microbiota diversity (Biagini et al. 2020), with the potential for contribution to further iterations of *in vitro* GI models. Through the use of either polysaccharide gels, gelatin, or mucin microcosms, the addition of these structures likely allows bacteria to form biofilms within the model, rather than just free-floating planktonic cells. Biofilms are considered to be a normal component of the intestinal environment, and some studies have suggested the biofilm lifestyle increases conjugation (Król et al. 2011, Stalder and Top 2016).

Other established models exist with the potential of contributing to our understanding of plasmid transmission in the human gut. With a similar structure to SHIME®, the SIMGI® Dynamic Intestinal Simulator (CIAL, Spain), adapted from Barroso et al. (2015), comprises five compartments spanning the stomach, small intestine, and three colonic reactors. The compartments are computer-controlled and can operate continuously or independently, and feature collection points for analysis of the reactor contents. The TNO *in vitro* model of the colon, TIM-2 (TNO, The Netherlands), designed by Minekus et al. (1999), consists of four compartments and incorporates peristalsis and a unique dialysate system to prevent accumulation of microbial metabolites (Venema 2015). Colonic models such as TIM-2 are often used in conjunction with upper GI models, such as TIM-1 (TNO, The Netherlands) or The Smallest Intestine (TSI; Cieplak et al. 2018). TIM-1 has been used to complement TIM-2 (Keller et al. 2019) and other colonic models, such as the single-vessel Artificial Colon (ARCOL; Applikon, The Netherlands; Blanquet-Diot et al. 2012). Despite not having yet been used to study conjugation, these GI tract models could be used to address questions relating to plasmid transmission.

A common and important factor amongst these models is that they attempt to recreate the stresses of earlier digestive processes, which better emulates the state of bacteria and compounds entering the colon. Inclusion of a low pH vessel, addition of bile and pancreatin at the appropriate stages further improves models, as these factors influence the composition of the microbiome, metabolic state, and even conjugation directly. For example, Aviv et al. (2016) found 1% bile reduced conjugation levels *in vitro*. Inclusion of mucin or mucus-like structures further adds to the physiological relevance of models and increases the translatability of results. When deciding on a model, each of these factors should be considered, and wherever possible incorporated.

There is no current standard *in vitro* model, due to different setups and lack of direct comparison between models. Community stability is difficult to ensure and compare, though validation studies investigating the stability of metabolic profiles and community composition have been performed and support the models discussed (Rajilić-Stojanović et al. 2010, Zihler Berner et al. 2013, van de Wiele et al. 2015, Liu et al. 2018, Biagini et al. 2020), with many using a high-throughput phylogenetic microarray, the Human Intestinal Tract Chip (HITChip; Rajilić-Stojanović et al. 2010, Zihler Berner et al. 2013, van de Wiele et al. 2015). Reproducibility within a model is also a key design concern made difficult by the complex and variable nature of microbiota and host factors, but can be approached with reductionist simplifications of both components (Elzinga et al. 2019). Defined microbial communities, as discussed earlier, have to date predominantly been used in colonizing animal models, with little but increasing application in *in vitro* systems (Elzinga et al. 2019).

### Host factors in *in vitro* models

Routinely absent in *in vitro* models due to the complexity of their incorporation are factors such as host–microbe cross-talk and immune modulation, which create a selectively pressured plastic environment for microbes. Host-derived factors such as cells, bile salts, antimicrobial peptides, and inflammatory mediators are sources of stress for bacteria and can modulate bacterial populations. As described previously, induced inflammation in streptomycin-treated mice has been observed to vastly increase conjugation efficiency of an IncI1 plasmid between *S*. Typhimurium and *E. coli* by initiating a rapid bloom of both populations (Stecher et al. 2012). Alternatively, host-derived factors have also been observed to directly inhibit conjugation, with Machado and Sommer (2014) identifying a host cell-secreted peptide-based factor that inhibited conjugation of an ESBL plasmid between *E. coli* clinical isolates in coculture with Caco-2 cells for 2 hours. The interplay between host and microbial elements of the microbiome is highly complex, and the effects on bacterial conjugation remain poorly understood, highlighting the necessity of research in this area.

The adenocarcinomal epithelial cell lines Caco-2 and HT29 are often used in host–microbiome studies, however, cocultures of cell lines with microbial populations are difficult to maintain in the long term, likely due to microbial cytotoxicity (van den Abbeele et al. 2009) and anaerobic environments (Ulluwishewa et al. 2015). Intestinal epithelial organoids, which have otherwise emerged as promising models due to their physiological structure, mimicking important features such as microbial product gradients (Wang et al. 2018, Costa and Ahluwalia 2019), encounter similar issues relating to oxygen availability. Leslie et al. (2015) successfully cultured *Clostridioides difficile* in an intestinal organoid model but detected up to 15% oxygen concentrations in the lumen, at which many anaerobic strains would be unable to persist.

Microfluidic, or ‘organ-on-a-chip’ models address the limitations of microbial toxicity and oxygen availability, by physically separating the cultures whilst allowing metabolite exchange and bacterial adhesion via semipermeable membranous structures. Systems such as the Host–Microbiome Interaction (HMI^TM^; Marzorati et al. 2014) and Host–Microbial Cross-talk (HuMiX; Shah et al. 2016) can increase viability of Caco-2 cells in coculture with microbial communities compared with direct contact and Transwell® membrane-separated models, respectively. Though both developed with a focus on the host, these methods providing longer viability of host cells in coculture present additional possibilities for investigating the microbiota *in vitro*, including potential use for studying impact of host factors on plasmid dynamics.

To date, these models have made little contribution to our understanding of plasmid dynamics within the gut. However, we think that their ability to simulate the microbiome environment, the relative ease with which these models can be manipulated, and their ability to reduce our reliance on animal models all make them good candidates to study plasmid interactions. As mentioned above, models which include some form of mucus-like structures, allowing for the formation of bacterial biofilms, are important to include in models used to study conjugation. We anticipate that over the next decade *in vitro* models will be refined and used to substantially expand our understanding of plasmid dynamics within the GI microbiome.

## Conclusions

Investigating conjugation in the human gut by direct observation is by far the best approach for understanding how bacteria behave and evolve in the real world, identifying which plasmids are of immediate threat to human health, and providing valuable insight into their properties and dissemination. For example, screening and surveillance of novel phenotypes from clinical isolates has led to multiple identifications of *K. pneumoniae* strains harbouring both carbapenemase-producing and virulence plasmids, resulting in the emergence of carbapenem resistant or MDR hypervirulent *Klebsiella* (Lam et al. 2019, Jin et al. 2021, Yang et al. 2021, Zhu et al. 2022). However, key limitations of this research approach include the lack of ability to ethically control most variables, requiring researchers to wait for these stochastic events to be uncovered by surveillance, as well as the limited access to the various internal environments.

Many of the limitations of *in situ* approaches are overcome by the use of experimental *in vivo* and *in vitro* models, which allow for greater manipulation of variables and monitoring of different locations. *In vivo* models benefit from aspects of immunity and GI structure, with murine models dominating the field from proof-of-concept to human GI simulating experiments. Comparisons with simplified *in vitro* liquid and agar mating experiments demonstrate cases where there is a discrepancy in observed plasmid dynamics, which gives insight for both identifying potential roles of the host, as well as better understanding of plasmid biology. *In vivo* models have clearly demonstrated that plasmid conjugation occurs in the gut microbiome, can occur inter- or intraspecies, and that density of donors and recipients is a key variable in conjugation frequency.

The increasing accuracy of highly specialized human-based *in vitro* models has expanded substantially in recent years. While still relatively rare, there are some important studies which have used *in vitro* models of the gut microbiota to study plasmid dynamics. These studies have clearly demonstrated conjugation of AMR plasmids to microbiota members *in vitro* (Haug et al. 2011, Smet et al. 2011, Machado and Sommer 2014, Lambrecht et al. 2019, 2021), both with (Smet et al. 2011, Lambrecht et al. 2021) and without (Haug et al. 2011, Machado and Sommer 2014, Lambrecht et al. 2019) antibiotic administration. Models which incorporate host cells have shown that host-derived factors can impact the rate of conjugation (Machado and Sommer 2014). In our opinion, these specialized *in vitro* models are still at an early stage in terms of how they have been used to study plasmid dynamics, but show great potential for allowing detailed experiments to uncover plasmid–microbiome dynamics and evolution. Despite a lack of experimental assessment on how these models compare against other approaches, such as murine models, in their translatability to studying plasmid conjugation, their success in related fields show their vast potential in AMR research.

The three approaches used to explore plasmid dynamics within the microbiome have contributed important and unique aspects to our understanding. *In situ* studies are critical in maintaining an up-to-date understanding of how bacteria are evolving in the clinic. Combined with genomics and bioinformatics, they have been used to identify newly mobilizable resistance genes, worrying combinations of ARGs, and convergence of AMR and virulence properties. *In vivo* approaches have contributed the most to our understanding of plasmid dynamics within the microbiome. They have been used to demonstrate that conjugation can occur at much higher levels in the gut compared to simple *in vitro* studies, and that bacterial cell density is a key factor in this. They have shown that certain plasmids (e.g. IncI2) seem to conjugate at high levels in the gut compared to other Inc groups, and that bacterial biology, such as motility, can influence conjugation. They have also highlighted the importance of host factors, such as inflammation, host genetic background, diet, and evolutionary bottlenecks resulting from intestinal reseeding all contribute to rates of conjugation. Studies using *Drosophila* and *Galleria* have shown that nonrodent animal models can be a good alternative to murine models. Finally, *in vitro* models have been used to demonstrate that factors such as pH, oxygen, bile, and microbial supernatants can all influence conjugation. Interestingly, *in vitro* studies also indicate an important role for mucin/polysaccharide/gelatin matrices in conjugation, potentially because they afford the bacteria a structure on which to establish close prolonged contact in a biofilm lifestyle.

There are still many unanswered questions about the fundamental biology of plasmid dynamics. As our understanding of these processes expands, our use of these models will adapt. For example, the recent paper by Low et al. (2022) demonstrated the importance of recipient cell receptors for conjugation, and the specificity of plasmid types for receptor types. This knowledge can be used in developing models to study plasmid transmission, e.g. using suitable recipients expressing the correct receptors, will increase conjugation frequencies. Furthermore, the increase in abundance of data from *in situ, in vivo*, and *in vitro* studies will benefit the development of *in silico* models used to simulate rate and incidence of conjugation in the human gut, as reviewed by Ott and Mellata (2022). The continued development of the discussed approaches will in turn further our collective understanding of the significance of the human gut microbiome on the dynamics of AMR plasmids, and their contribution to the emergence and prevalence of clinically important pathogens.
